# Supplementary material for: Chromothripsis during telomere crisis is independent of NHEJ, and consistent with a replicative origin
Source: Genome Res. 2019 May;29(5):737–49. doi: 10.1101/gr.240705.118 (PMC6499312; doi:10.1101/gr.240705.118)
Supplement: Supplemental Material [file supp_gr.240705.118_Supplemental_file_1.zip › contigs/annotated_contigs/DB49/contig.5.DB49_length_1155_mean_cov_12.1523809524.docx]

**DB49_length_1155_mean_cov_12.1523809524**

```
AAAAGAACCGAAACACTCCAGCAGAAG|AAACAAAAGCAAAAAACAATAACAAA|AAAACAAAACAAAAAACAAAAA|AATAATAAAAA
                            >chrX:9680339-9680387 - E=7e-02 p=0e+00 >chrY:13542943-135
ACACGGACCAGAAAGTGTCATCCTACTGACAAATTGAGAAGACAAGGCAGTTTGTCTTAGCTGGTCTCTGTGTGGCAAAGTCTTTTTTG
43354 + E=7e-204 p=2e-02
AGAATTCATATGTAAAACTGGCCTCATCCAACTTATGTGTAGGGCTCAAATTACATTACGTGCACAGGTTAGGGATCCCAAAGTTCTAA

ACCTACCATAAAAGTGTCCGTGCATAGAAGTATCCCTAGAACACTTAGTAGATGCTAATTCAAAAAATCTCTAATAGACACTCCTGATC

CCAGACTGCATAAAGTCTCAAAGGTAAAGCCAAGTGAATCTGAACTTAAAATAAGAAGAAATATCCCATAAGCAAAATTAACAGATACA

ACAAAGAATGGGATTAGATGTCTA|TC|TAGAACCACTAGATAGACACTATAAAATATGTATTTTCAAAGGGACTAGAGACAATTTTTA
                           >chr4:103872592-103872689 + E=2e-46
AAAATTGGAGACATGAGAAGAAGAAATTACTTGAAA|TACAAAATTAGCCAGGCATGGTAGTGCC|TGCCTCTAATCCCAGCTACTCGG
                                                                 >chr3:155652876-155653
GAGGCTGAGGCAGGAGAATCACTTGTAACAGGAGGCAGAGGCTGAAGTGAACCAAGATTGTGCCACTGCACTCCAGCCTGGGCGACAGA
117 - E=7e-130
GCAAGACAGACTCCGTCTCAAGAAAAAAAAGCAACATCATAGAACATTCAAAGATTTAAATAATAAAAACAAAAGCATCTAAAAAGCAA

AAGCCACATCCTCCTACTCTTAATATCAAGCTTCCAAAAA|G|ATGTGCAAAGATATAAGTGATTATTACAACTCTCTTTTCACTTAAT
                                          >chr3:155652143-155652463 - E=2e-180
GTACTGGGTCGAAGAGTATCCCCAAAACTTCATGCCCACCCAGAATCTCTGGGGAGAAAAAGGGTCTTTGCAAATGTAATCAAGTTAAA

ATGAAGTAATACTGGATTAGGATGGGCTCTAATCCAATGACTGGTATCCTTCCAAGTGCTGGGAAATTTGGATACACAGATACCCACAG

GGGAATGAGGGTCATGTGAACACAAAGACAGACTGGACTATGCTGCCACCACTCGATAGTAGGAAGAAGCAAGGAAGGATCCTCCCCTA

GAGTCTT
```
